# Evaluating Expert-by-Experience Input in Psychiatric Training for Mental Health and Special Educational Needs Tribunal Members

**DOI:** 10.1192/bjo.2026.11182

**Published:** 2026-06-30

**Authors:** Mohammed Alkahlout, Ella Cheney, Rachel Moody, Carolyn Fyall, Joan Rutherford

**Affiliations:** 1 King’s College London, London, United Kingdom; 2 East London NHS Foundation Trust, London, United Kingdom; 3 London, United Kingdom; 4 First Tier Tribunal, London, United Kingdom; 5 South London and Maudsley Foundation Trust, London, United Kingdom

## Abstract

**Aims:**

As guided by the General Medical Council and the Royal College of Psychiatrists, the inclusion of experts by experience is a required component of British undergraduate medical education and postgraduate psychiatry training. However, this has not been formally applied to the training of Mental Health Tribunal (MHT) and Special Educational Needs and Disability (SEND) Tribunal members who are advised on reasonable adjustments for patients and appellants through the Equal Treatment Bench Book, including guidance on Autism Spectrum Disorder (ASD).

This study aims to evaluate the impact of expert-patient input on tribunal members’ understanding of ASD following a dedicated training programme. We hypothesise that the training day will increase participants’ confidence regarding reasonable adjustments for patients with ASD and will be perceived as novel and valuable.

**Methods:**

A multidisciplinary training day was delivered to MHT and SEND Tribunal members in England, jointly by clinicians and experts by experience. The training offered a multimodal overview of ASD and its treatment, and how reasonable adjustments can be made in tribunal hearings.

To evaluate the session, data was collected via online post-session feedback questionnaires. Items were selected to assess participants’ confidence with regard to the training’s learning outcomes, the perceived novelty and value of expert-by-experience involvement, and participants’ willingness to receive a follow-up email six weeks post-training to support consideration of reasonable adjustments.

**Results:**

The training programme was attended by 54 MHT and SEND Tribunal members, of which 24 were judges, 13 were consultant psychiatrists, and 17 were other specialists involved in the care of patients with ASD.

Ninety-four per cent of respondents reported feeling confident after the training session, an increase from retrospectively reported pre-session confidence of 50% (n=18). Over half had no prior exposure to expert-by-experience training (55%, n=47), while a large majority thought MHT and SEND tribunal training should include more input from experts by experience (89%, n=46). 68% of participants (n=45) opted to receive an email six weeks post-training to be prompted to continue to make reasonable adjustments for patients with ASD.

**Conclusion:**

The findings reinforce the effectiveness of expert-by-experience input and highlight its lack of incorporation to date in tribunal training. The willingness of participants to receive a follow-up email six weeks post-training suggests an openness to reflection. Further work should explore how such involvement can be sustainably embedded within tribunal training to support awareness of mental health conditions, including ASD, and the implementation of reasonable adjustments in hearings.

